# Thermal performance evaluation of phase changing materials in double glazing units for office buildings in Egypt

**DOI:** 10.1038/s41598-025-88206-x

**Published:** 2025-02-06

**Authors:** Omar Abdel-Rahman, El Rouby, Mohamed Moemen Afify

**Affiliations:** 1 Architecture Engineering Department, Faculty of Engineering, New Giza University, Cairo, Egypt; 2 Prof. of Architecture & Environmental Design Department, Faculty of Engineering, Cairo University, Cairo, Egypt

**Keywords:** Environmental sciences, Energy science and technology, Engineering

## Abstract

Nowadays glass curtain walls are used widely in modern buildings as they offer a significant aesthetic appearance and provide the building with natural lighting. However, their poor thermal resistance leads to excessive usage of mechanical systems to achieve thermal comfort which in turn increases the energy consumption in buildings. The proper integration of the unique properties of the PCM could effectively enhance the thermal performance of buildings therefore, the main objective of this research is to examine and validate the effect of using phase change material (PCM) on the building thermal performance especially when integrated with double glazing unit (DGU). The research examined comprehensively the effect of using PCM with DGU by undergoing a field experiment and comparing the results with a simulation model using Design Builder (DB) -energy plus simulation tool. After validating the simulation model, DB was used to examine the effect of using PCM double glazing unit with a multi-story office building in three different climatic regions in Egypt covering humid, mild, and hot arid regions. The results showed that using petroleum jelly as a PCM with DGUs will lead to a significant effect in reducing the usage of mechanical systems in cooling the building spaces by 8.91%, 8.62%, and 8.07% in Cairo, Alexandria, and Aswan consecutively from the total yearly cooling electric consumption.

## Introduction

Building and construction fields play a pivotal role in energy consumption globally, accounting for approximately 40% of the world’s energy consumption^1^. This demand is predicted to be increased to reach 58% of the world total energy consumption by 2050^2^. Building envelopes are one of the main factors for energy consumption in buildings, especially for hot climate regions. Building geometry ratios, finishing materials, orientation, and window to wall ratio (WWR), are all listed as the main envelope features that if tracked can help in reducing the energy consumption in buildings^3^. About 40% of the energy losses in buildings are attributed to buildings’ exterior facades^4^. More than 50% of the total energy consumption in buildings is due to the heating, ventilation, and air conditioning (HVAC)^5,6^. In Egypt, according to the Egyptian electricity holding company latest annual report 2020/2021, building sector is a significant contributor in energy consumption. Residential buildings are responsible for 40.5%, commercial buildings for 13%, governmental buildings for 4.8%^7,8^.

Glass curtain walls (GCW) are a common feature in most modern buildings nowadays. The architectural aesthetics appearance of glass curtain walls helped in spreading its usage especially with office buildings. However, traditional buildings still have the lead in indoor thermal comfort due to their higher thermal resistance if compared to the GCW buildings. That results in an increase in GCW buildings energy consumption as they rely heavily on heating, ventilation, and air conditioning (HVAC) systems to maintain indoor thermal comfort in such buildings^9,10^. In Egypt, as a result of the high temperature and intense solar radiation especially in summer, air conditioning devices are used extensively, which in turn has increased energy consumption in building to achieve indoor human thermal comfort^11^.

Windows have a significant effect on reducing energy consumption in buildings. They contribute to about 60% of buildings’ total energy consumption^12^ Replacing single glazing windows with double glazing windows could lead to savings of 39 to 53% of energy in commercial buildings. As a result, glazing technologies gained considerable interest from researchers. Vacuum glazing, smart glazing, low-emissivity coated (low-e) glazing, and PCM glazing, are all glazing technologies capable of providing high thermal resistance for GCW^13^. Cuce et al. studied through a comparative experimental investigation the thermal performance of three glazing technologies, heat insulation solar glass, vacuum tube window, and solar pond window. The results showed that the vacuum tube window has the highest thermal resistance with a U-value of 0.4 W/m^2^K. Heat insulation solar glass and solar pond window, showed also a promising U-value compared to traditional glazing window^14^. The U-value of vacuum glazing could be optimized to 0.2 W/m^2^K by integrating with low-e coatings^15^.

Phase change materials (PCMs) are introduced as an effective solution in reducing energy consumption in buildings. Integrating PCM with buildings roof and walls helps in decreasing the annual energy consumption in buildings by 25.7–47.1% according to the thicknesses of the PCM used^16^. Jaradat et al.^17^ studied residential buildings envelopes when enhanced with bio-based PCM. The findings showed a significant improvement in the building energy consumption, the heating energy was decreased by 34.38%, and cooling energy was decreased by 23.33%. Mohseni and Tang^18^ experimentally examined the thermal performance of integrating PCM into different building elements. The results showed a clear reduction in the heating and cooling energy consumption by 23% and 12% when increasing the thickness of PCM from 5 mm to 10 mm, respectively. Also using PCM with melting temperature of 21 °C in winter, and 25 °C in summer have the best performance in reducing energy consumption. Zhou and Razaqpur performed a comparison between a traditional static Trombe wall and a dynamic Trombe wall integrated with PCM. The results showed 20% thermal efficiency improvement in the dynamic Trombe wall in comparison to the traditional one^19^. Cuce et al. examined various roofing options of integrating either PCM or polyurethane foam insulation in hot arid and warm humid climatic zones in India. The results showed that the acrylic PCM integrated roof had the best annual energy cost savings with the shortest payback period^20^. Another study examined experimentally and numerically the impact of incorporating different types of PCMs into the building roofs. It also explored the PCMs placement within the roof structure, considering locations on the interior, exterior, and middle layer of the roof. The results showed that in hot arid and warm-temperate regions in India, the hydrated salt PCM have the best overall reduction in energy cost especially when placed above the roof^21^.

Also integrating PCMs with double-glazed windows showed a significant reduction in energy consumption in buildings. Adding PCM to double-glazing units (DGU) results in an improvement in its thermal resistance when compared to air-filled double-glazing units in dry arid regions^22^. Shu Zhang et al.^23^ studied the integration of PCM and silica aerogel to low-e glass in multiple glazing roof. The research stated that the integration of silica aerogel and low-e glass can remarkably improve the thermal performance of PCM filled glazing roof, also the saving of energy in summer and winter was recorded 14.08% and 33.74%, respectively. Another research conducted by Dong Li et al.^24^ studied experimentally the effect of PCM with glazed roof, it included the PCM melting temperature, the PCM layer thickness, and the angle of slope of the glazed roof and their effects on the energy consumption. The results showed that the incorporation of PCM of melting point of 32 °C instead of air in roof glazing unit could significantly reduce the energy consumption by 47.5%. The research also revealed the effect of the PCM thickness and the slope of the glazing roof on improving the indoor thermal environment.

Accordingly, the incorporation of PCMs to the glazing units can effectively decrease the excessive use of air conditioning in buildings leading to a reduction in the building energy consumption. However, very few research has investigated the usage of Vaseline as PCM with DGU. In this work, the researchers address this gap by studying experimentally and numerically the effect of integrating Vaseline as a PCM into DGU in comparable to traditional DGU filled with air. After validating the results, a simulation for a multistory office building model was investigated in humid, mild, and hot arid regions in Egypt. This research contributes significantly to the field of PCM applications in the building envelope, highlighting the effective integration of PCMs into DGU.

## Methodology

**Fig. 1:**
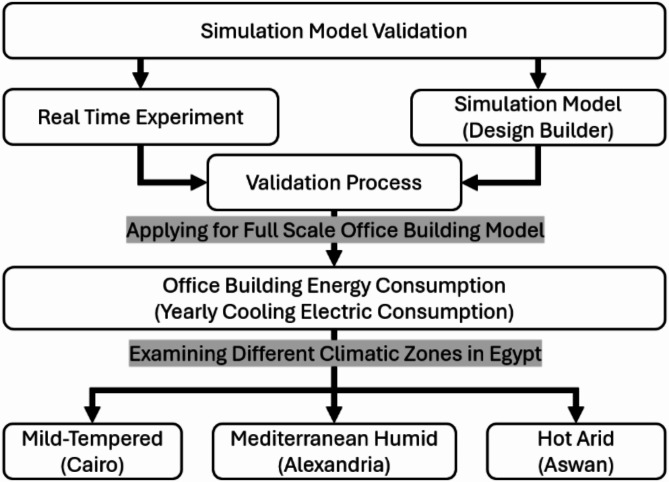
Flow chart of the methodology of the study.

The aim of this research is to study the thermal effect of integrating PCM to DGU in Egypt. Firstly, two identical experimental boxes were constructed to investigate and compare the thermal efficiency of the traditional DGU filled with air and the second DGU filled with PCM. Then, two similar boxes were built using Design builder (DB) software version 7.0.2.004. DB is a building performance simulation tool that uses Energy Plus 9.4. as a simulation engine with a user-friendly interface^25,26^. Both the experimental and simulation results were then compared to validate the accuracy for a real scale office building model. (Fig. 1)

### Experimental setup

**Fig. 2:**
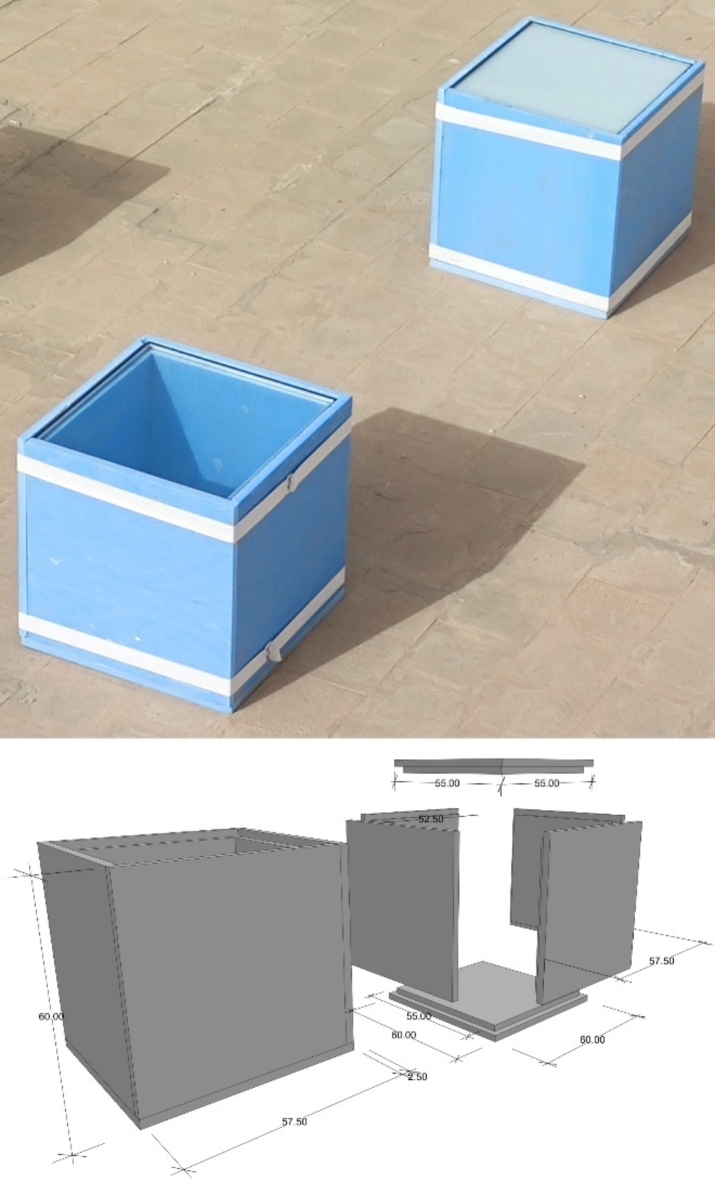
(Up) Real image for the experimental boxes with the DGUs. (Down) Schematic drawing with dimensions (cm) for the experimental boxes.

**Fig. 3:**
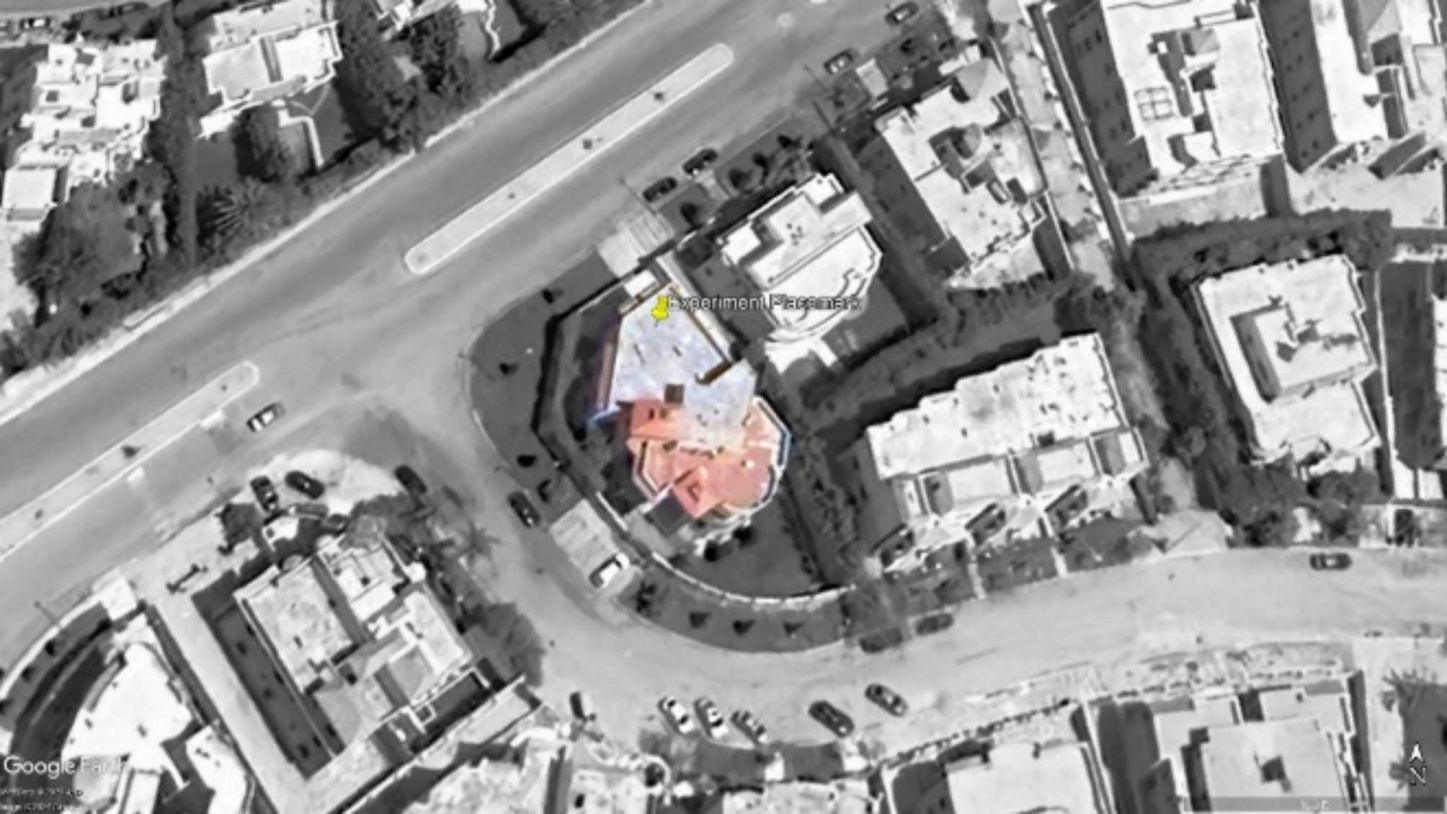
Experiment location google earth map- latitude 30° 1’31.71” North, and longitude 30°59’32.66” East- (Generated from Google Earth Pro and edited by the author).

Two laboratory room models were constructed with dimensions of 600 mm x 600 mm x 600 mm. The two cubes have five sides of extruded polystyrene thermal insulation boards of 50 mm thickness (Fig. 2). The top horizontal side is once covered with the traditional DGU filled with air, and the second is covered with DGU filled with the PCM. The experiment was conducted on the roof a residential building located in El-Shiekh Zayed, Cairo, Egypt, lies at latitude 30° 1’31.71” North, and longitude 30°59’32.66” East (Fig. 3). The environmental conditions of the experiment were calculated from the data derived from a datalogger that was placed in a shaded area near the experimental boxes. The average temperature during the experiment was calculated to be 18.12 °C with a recorded maximum temperature of 28.1 °C and minimum temperature of 11.6 °C and the average relative humidity was calculated to be 62.3% with a recorded maximum humidity of 88.5% and minimum of 29.4%.

**Fig. 4:**
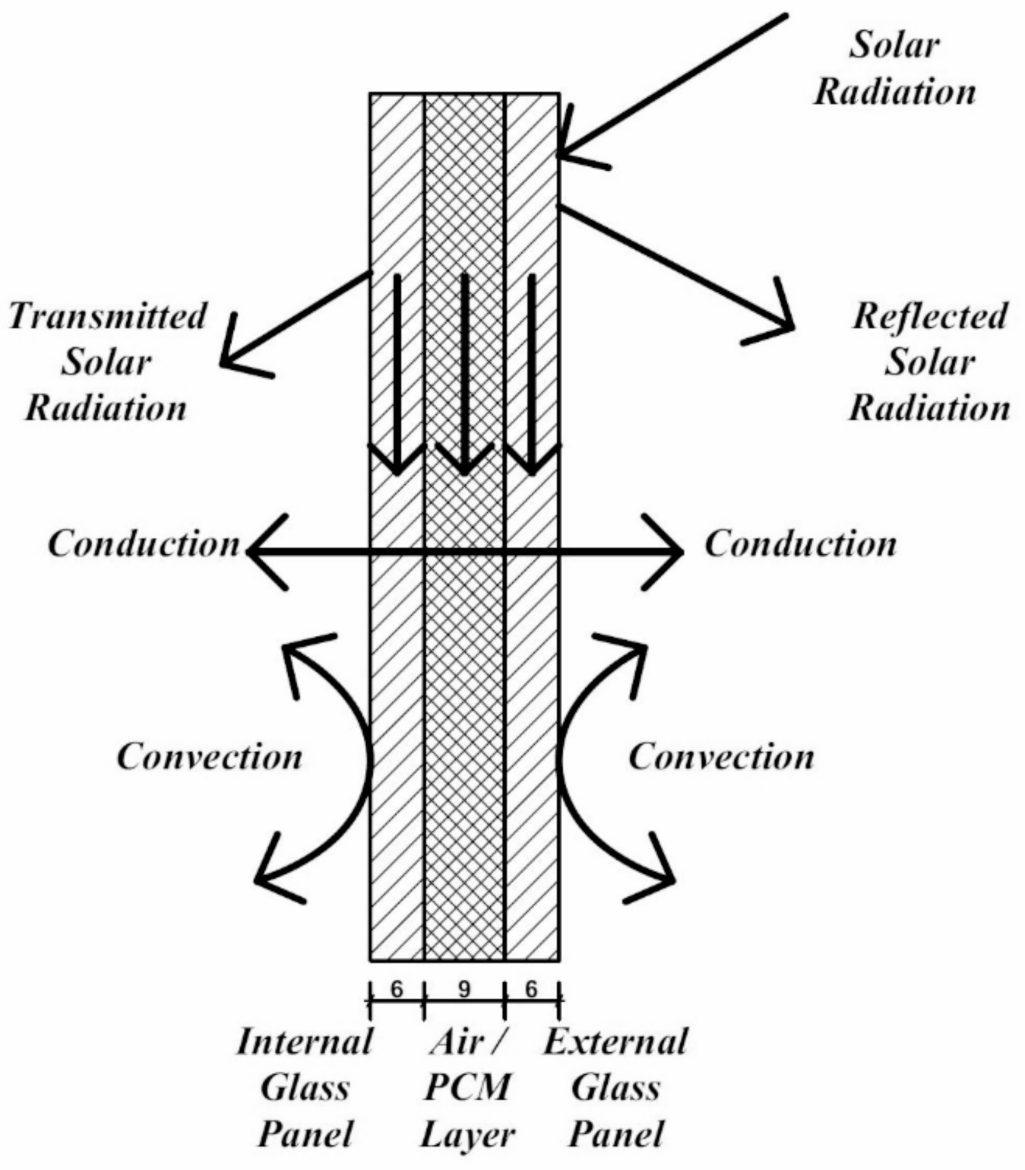
Schematic drawing (mm) for the DGU cross-section.

**Fig. 5:**
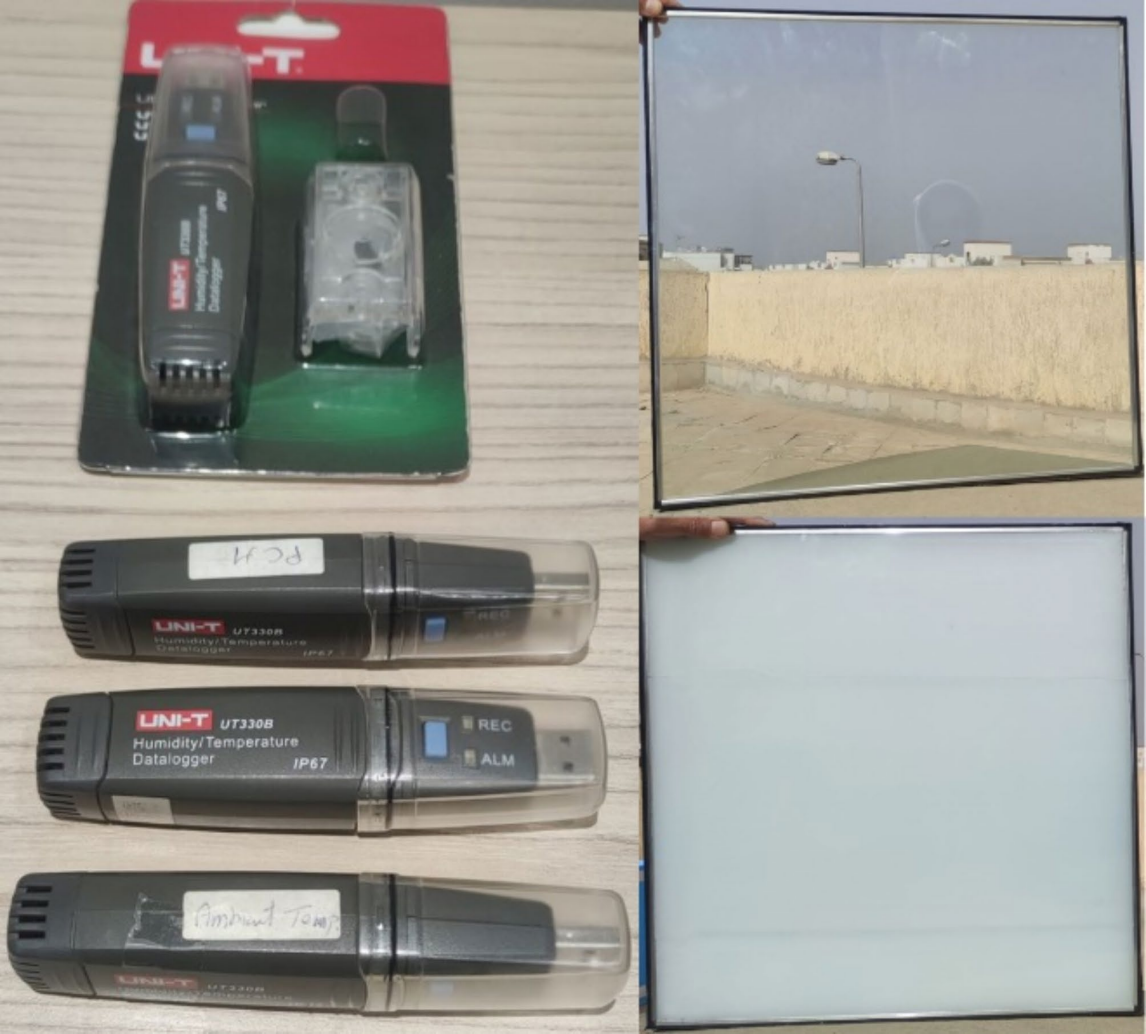
(Left) set of dataloggers used through the experiment. (Up right) DGU with air gap. (Down right) DGU with PCM in solid state.

**Fig. 6:**
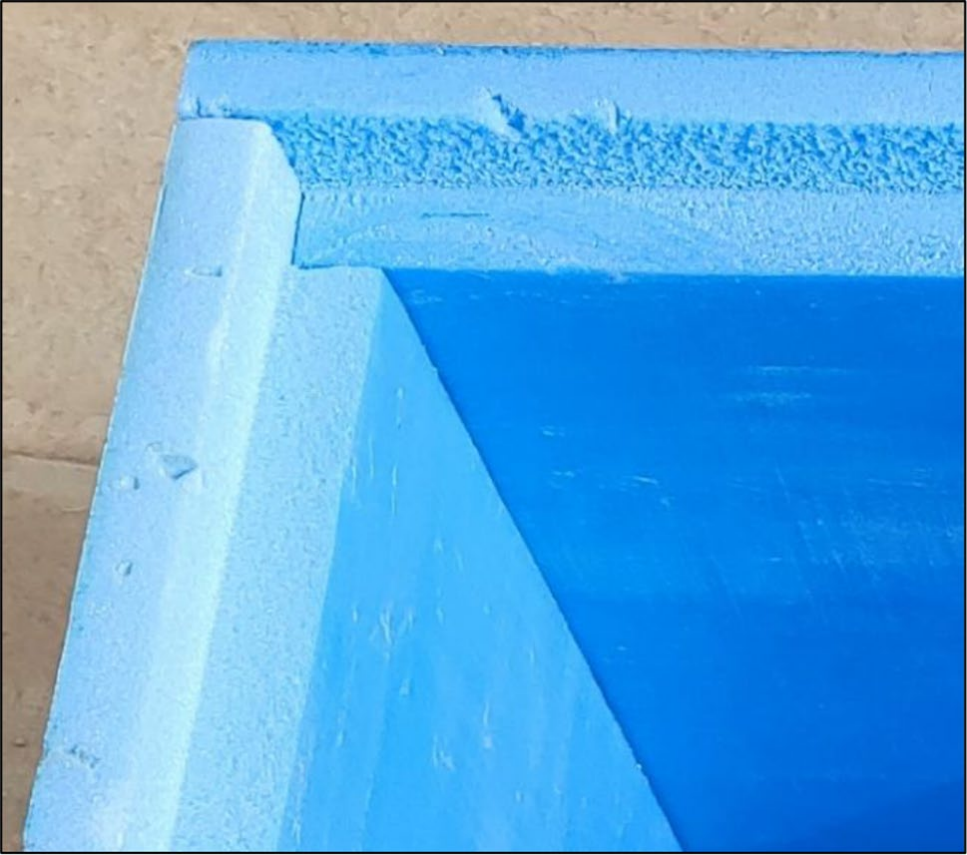
Half-lap Joint connection of the box’s sides.

Each of the two DGU consists of two glass panels with dimensions of 550 mm x 550 mm. The traditional DGU glass panel thickness is 6 mm with an air cavity of 9 mm with a total thickness of 21 mm (Fig. 4). The PCM DGU consists of two glass panels of 6 mm thickness with an injected PCM of 9 mm with a total thickness of 21 mm (Fig. 5). Four edges of the PCM DGU were well sealed with epoxy, and a 5 mm hole was drilled from one side to inject the PCM after which that hole was sealed. To decrease air infiltration, the edges of the four sides and the base of the boxes were connected to each other with a half-lap joint (Fig. 6) to enhance the tightness and decrease the air infiltration through the edges^27^. The boxes were also tied up with two rubber bands for the same reason. (Fig. 2)

Three identical UT330-USB Series Datalogger were used during the experiment with measurement range of -40 °C ~ 80 °C, accuracy level of ± 0.5 °C for the temperature range 0 °C ~ 40 °C, and resolution of 0.1 °C (Fig. 5). The dataloggers were set to record the temperature every 60 min for four consecutive days. The Dataloggers were placed in each box – Traditional_DGU, and PCM_DGU - and another one was placed in shaded area to measure the ambient temperature. Before conducting the experiment, the three dataloggers were left in the same environmental condition for calibration. The average recorded temperature was 21.53 °C with a standard deviation of 0.31 °C and an accuracy level of 1.4%.

### Materials specification

The materials used in the experiment were chosen carefully according to the availability in the local market. The extruded polystyrene thermal insulation boards were purchased from a well-known manufacturer in Egypt to ensure the unity and quality of all boards used in the experiment. The thermo-physical properties are listed in Table 1.

**Table 1:** Thermo-physical properties of experiment materials.

Material	Extruded Polystyrene^28^	Glass^13^
Thermal conductivity (W m^-1^ C^-1^)	0.0288-0.032 *	1
Average Density (kg m^-3^)	34-36	2400
Thickness (mm)	50	6

* Based on age of boards.

The process of choosing the appropriate PCM was challenging, Hussein Akeiber et al.^30^ stated four main criteria for the selection of the PCM. Thermodynamic properties, chemical properties, economic properties, and finally kinetic properties are the main selection criteria for the chosen PCM. Petroleum jelly (Vaseline) was chosen for the experiment as it meets the main physical and thermal properties suitable with Cairo’s weather conditions. Its availability and affordable price were one of the main keys in the selection process. Petroleum jelly (Vaseline) was used as PCM in many building applications to improve the indoor air temperature^31,32^. Table 2 shows the thermo-physical properties of Vaseline^33^. Although the wide range of Vaseline melting point^34^, the available Vaseline in local market has a melting point of 46 °C.

**Table 2:** Thermo-physical properties of used PCM.

PCM	Petroleum Jelly (Vaseline)
Thermal conductivity (W m^-1^ C^-1^)	0.18
Average Density (kg m^-3^)	850
Thickness (mm)	9
Melting Point (°C)	36 – 60^29^

The experiment was carried out for four consecutive days, from March 4th, 2024, to March 7th, 2024. The results were then extracted from the dataloggers to be analyzed.

### Design builder model

**Fig. 7:**
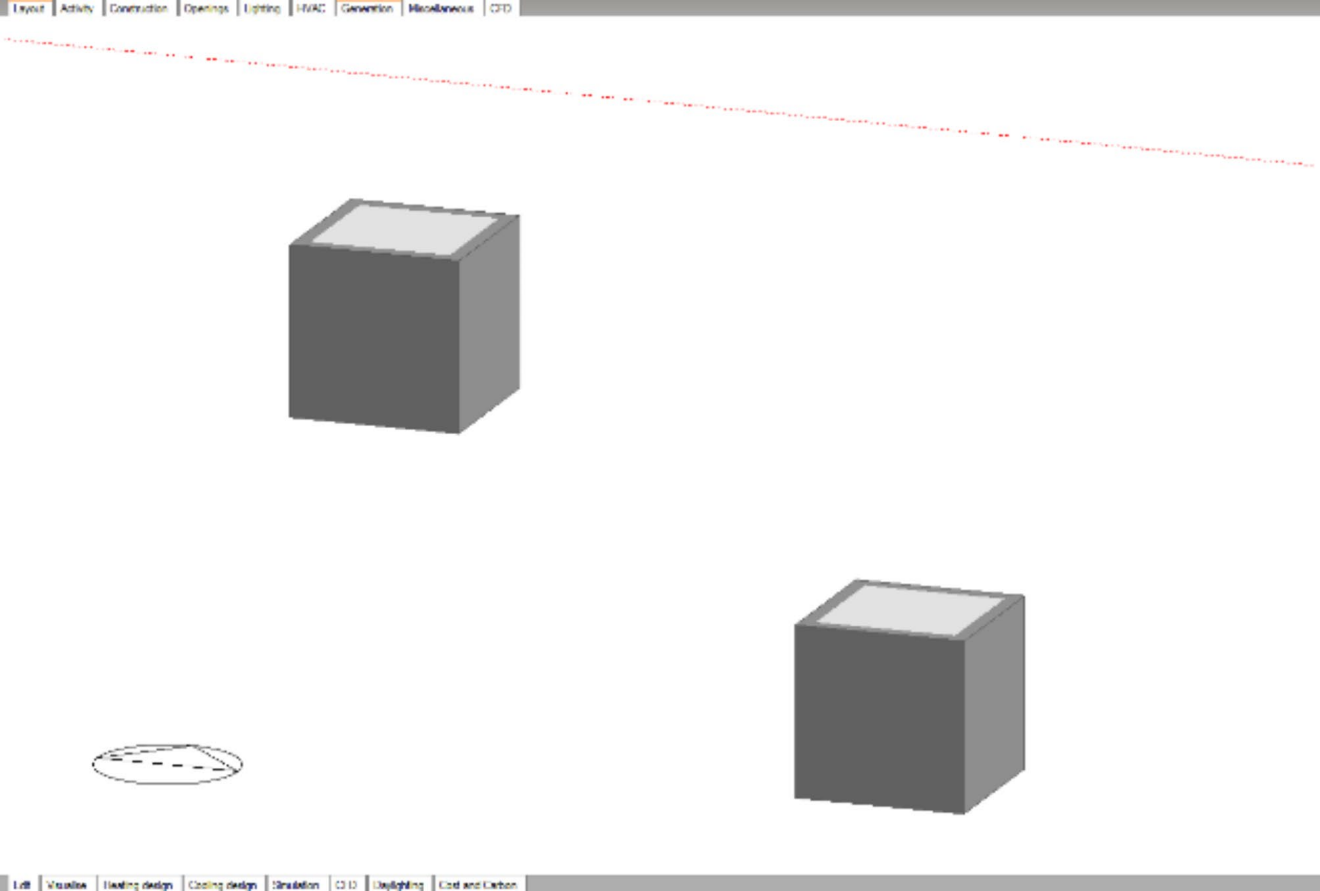
Validation models on Design Builder.

Three-dimensional models were generated using Design Builder software identical to those of the experimental boxes. The nearest weather station to the experiment location is at Sphinex international airport so, Cairo west weather file was uploaded to DB to get the most accurate weather data. Two boxes were generated having the same dimensions and materials as the experimental boxes. An extruded polystyrene of 50 mm thickness for base and four sides, and one box having traditional DGU for the top side, and the second one having PCM DGU for the top side. (Fig. 7)

### Model validation

**Fig. 8:**
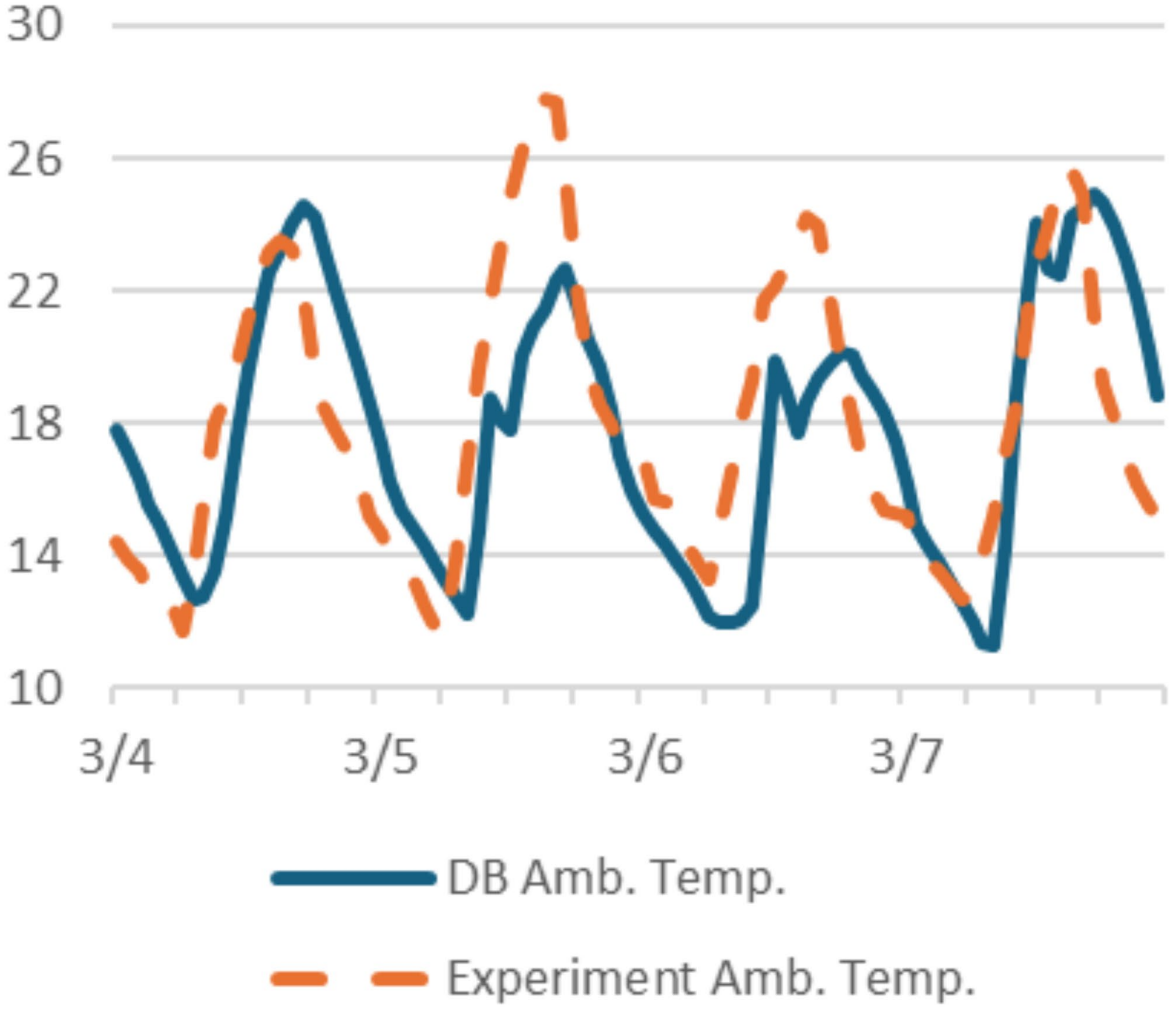
Comparing ambient temperature extracted from the experimental and simulation models for two boxes (4/3–7/3/2024).

To validate the numerical model, the boundary conditions were set in DB to replicate that of the experimental setup. Indoor conditions, materials thermos-physical properties, air tightness and infiltration rate, and outdoor conditions were adjusted to imitate the real time experiment. Finally, the simulation period was set in DB to replicate the same dates the experiment was conducted. The results were first extracted from the ambient temperature data logger to study the deviation between the recorded temperatures during the experiment and the outdoor temperature values of the DB simulated model. (Fig. 8) shows a strong correlation between the outdoor temperatures of the DB simulated model and the extracted data from the ambient temperature datalogger. The average deviation ranges were calculated using Eq. (1)^35^.

1 $$\:{Dev}_{\mathcal{i}}=\left|\frac{{T}_{exp\mathcal{i}}-\:{T}_{sim\mathcal{i}}}{{T}_{exp\mathcal{i}}}\right|*100\%$$

Where ($\:{Dev}_{\mathcal{i}}$) is the deviation at time $\:\mathcal{i}$, ($\:{T}_{exp\mathcal{i}}$) is the experiment average temperature, and ($\:{T}_{sim\mathcal{i}}$) is the simulation average temperature.

The results showed an average deviation of 2% between both the experimental and the simulated average outdoor temperature for the examined period, which is a valid percentage to continue examining the indoor temperature of the two boxes and comparing the results to validate the DB model.

**Fig. 9:**
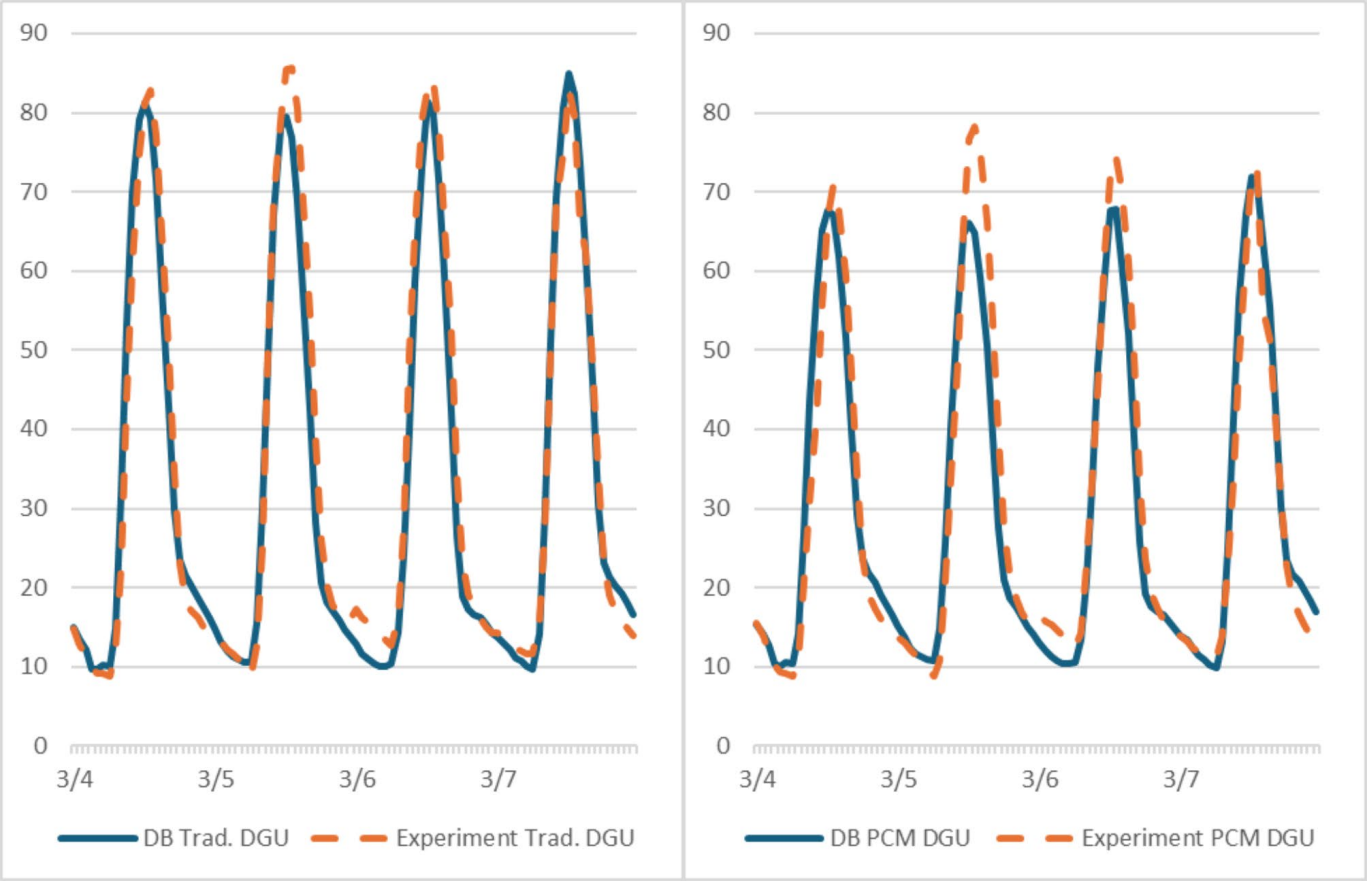
Comparing indoor temperature extracted from the experimental and simulation models for the two boxes (4/3–7/3/2024). (Left): Box with traditional DGU model. (Right): Box with PCM DGU model.

When comparing the results of the indoor temperature of the two boxes extracted from the data loggers with the results generated from the DB for the same period (Fig. 9), it showed that the average temperature deviation of the traditional DGU room, and that of the PCM DGU room were 2.57%, and 3.98%, respectively. Accordingly, the generated DB simulation model showed a reliable, valid, and accurate model that could be used for full-size building simulations.

### Location selection

To examine the thermal performance of the PCM double glazed curtain wall and its effect on the indoor thermal comfort in different climate regions, three different locations were selected based on the three main climatic zones in Egypt^36^. The three climate zones are:

(i) Delta and Cairo zone (Cairo governorate), as the capital with the highest population, Cairo is located at latitude 30.13° North, and longitude 31.4° East, and its elevation above sea level is 74 m. According to climate consultant 6.0, the average high temperature is 27.74 °C, and the average low temperature is 17.22 °C, with a mean temperature of 22.22 °C.
(ii) North Coast zone (Alexandria governorate), as a Mediterranean humid zone, Alexandria is located at latitude 31.2° North, and longitude 29.95° East, and its elevation above sea level is 7 m. According to climate consultant 6.0, the average high temperature is 24.89 °C, and the average low temperature is 16.09 °C, with a mean temperature of 20.37 °C.
(iii) Southern Egypt zone (Aswan governorate), as a hot arid zone. Aswan is located at latitude 23.97° North, and longitude 32.78° East, and its elevation above sea level is 194 m. According to climate consultant 6.0, the average high temperature is 32.76 °C, and the average low temperature is 19.67 °C, with a mean temperature of 26.17 °C^37^.

### Simulation study

**Fig. 10:**
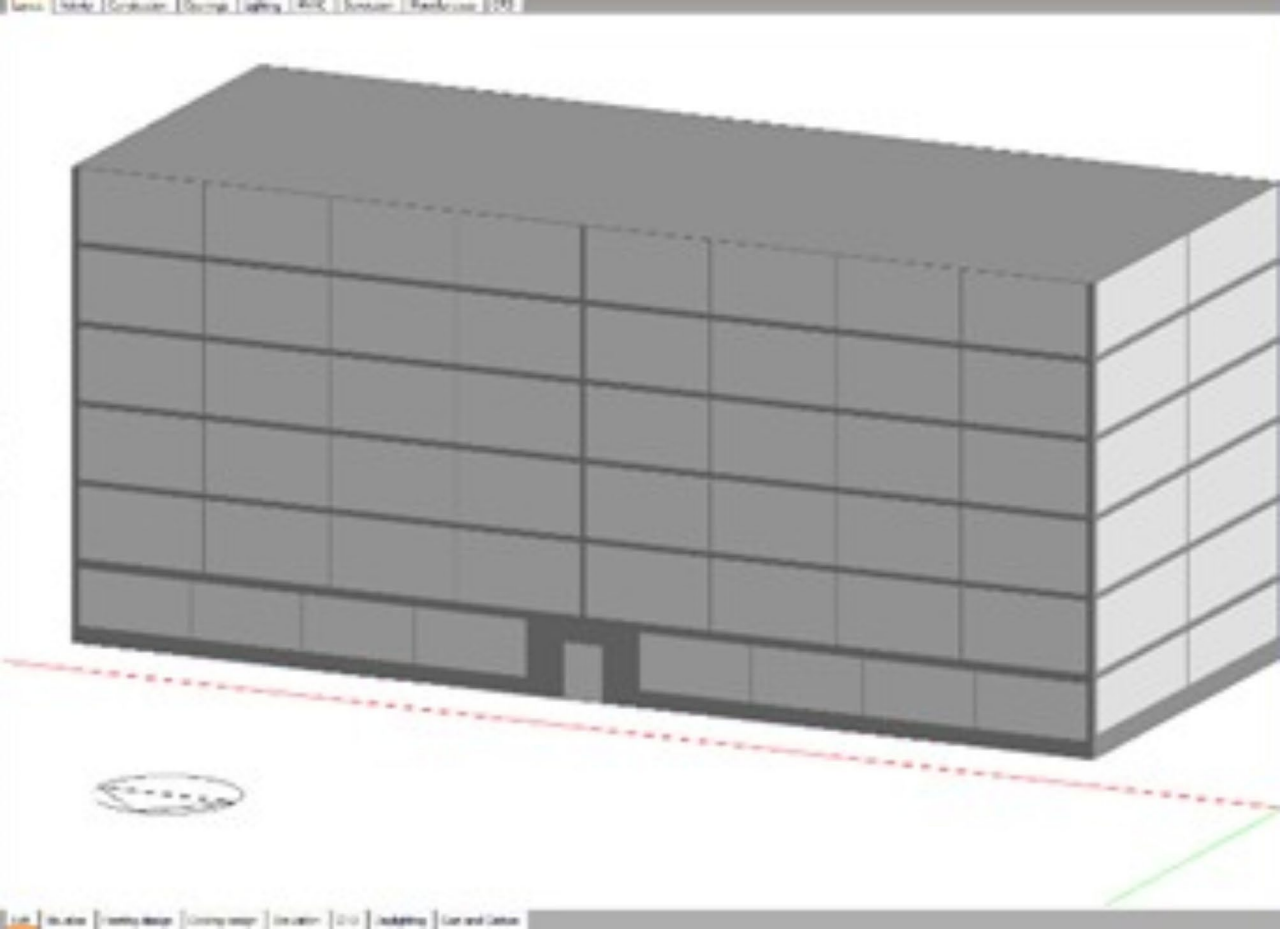
Typical six-story GCW office building modeled in Design Builder.

A typical six-story office building was conducted using DB software for the simulation study. As shown in (Fig. 10) the office building consists of ground and five typical floors, each of 3.5 m in height and a floor area of 1000 m^2^ (50 m length, and 20 m width). Traditional DGUs were used for all GCWs for the whole building in the base case. For the case studied, the PCM DGUs were implemented to the south and west building elevations with a percentage of 50% of the GCW building elevations. The reason for this is that the PCM at its solid state is translucent so, to give the building users a clear view for the building surroundings and not blocking the whole seen, the decision of implementing 50% only of the GCW with PCM DGU was taken. Table 3 shows the different building construction elements layers (floor, roof, and walls) according to the traditional construction building layers in Egypt, showing the thermo-physical properties of each building material. The office building operation schedule was set to five days working days per week (Sunday to Thursday), with working hours from 08:00 to 18:00 each day.

**Table 3:** Different building construction materials thermo-physical properties (as retrieved from Design Builder).

Item	Layers	Thermal Conductivity (W/m.K)	Density (kg/m3)	Specific Heat Capacity (J/kg.K)	U-Value (W/m2.K)
**External Wall**	Outermost Layer / Plaster (3 c.m.)	0.35	950	840	1.515
	Layer 01 / Brick (25 c.m.)	0.72	1920	840	
	Innermost Layer / Plaster (2 c.m.)	0.35	950	840	
**Roof Floor**	Outermost Layer / Roof Tiles (2 c.m.)	1.5	2100	1000	0.428
	Layer 01 / Mortar (2 c.m.)	0.4	1200	840	
	Layer 02 / Sand (6 c.m.)	1.5	2180	720	
	Layer 03 / Slope Concrete (7 c.m.)	0.29	850	840	
	Layer 04 / Extruded Polystyrene (5 c.m.)	0.034	35	1400	
	Layer 05 / Damp Proof Insulation (2 c.m.)	0.17	1050	1000	
	Layer 06 / Reinforced Concrete (15 c.m.)	1.9	2300	840	
	Innermost Layer / Plaster (2 c.m.)	0.35	950	840	
**Ground Floor**	Innermost Layer / Marble Tiles (2 c.m.)	2.9	2750	840	1.665
	Layer 01 / Mortar (2 c.m.)	0.4	1200	840	
	Layer 02 / Sand (6 c.m.)	1.5	2180	720	
	Layer 03 / Plain Concrete (10 c.m.)	1.4	2100	840	
	Layer 04 / Damp Proof Insulation (2 c.m.)	0.17	1050	1000	
	Outermost Layer / Plain Concrete (15 c.m.)	1.4	2100	840	
**Typical Floor**	Innermost Layer / Marble Tiles (2 c.m.)	2.9	2750	840	1.993
	Layer 01 / Mortar (2 c.m.)	0.4	1200	840	
	Layer 02 / Sand (6 c.m.)	1.5	2180	720	
	Layer 03 / Reinforced Concrete (15 c.m.)	1.9	2300	840	
	Outermost Layer / Plaster (2 c.m.)	0.35	950	840	

## Results and discussion

The process of investigating the energy saving of using the PCM double glazing unit was simulated using Design Builder. The total required electricity for the building cooling was calculated for the base case office building with traditional DGU and the examined office building implemented with 50% PCM double glazing unit for the three selected locations monthly. The yearly output was then calculated for both cases and the energy saving rate was calculated using the following Eq. (2):^25^

2 $$\:{\upeta\:}=\left|\frac{{Q}_{no\:PCM}-\:{Q}_{\:PCM}}{{Q}_{no\:PCM}}\right|*100\%$$

Where ($\:{\upeta\:}$) is the yearly energy saving rate, $\:{Q}_{no\:PCM}$ is the total yearly electric cooling consumption for the base case (building with traditional DGUs), and ($\:{Q}_{\:PCM}$) is the total yearly electric cooling consumption for the examined case (building with PCM double glazing units).

### Cairo location

**Fig. 11:**
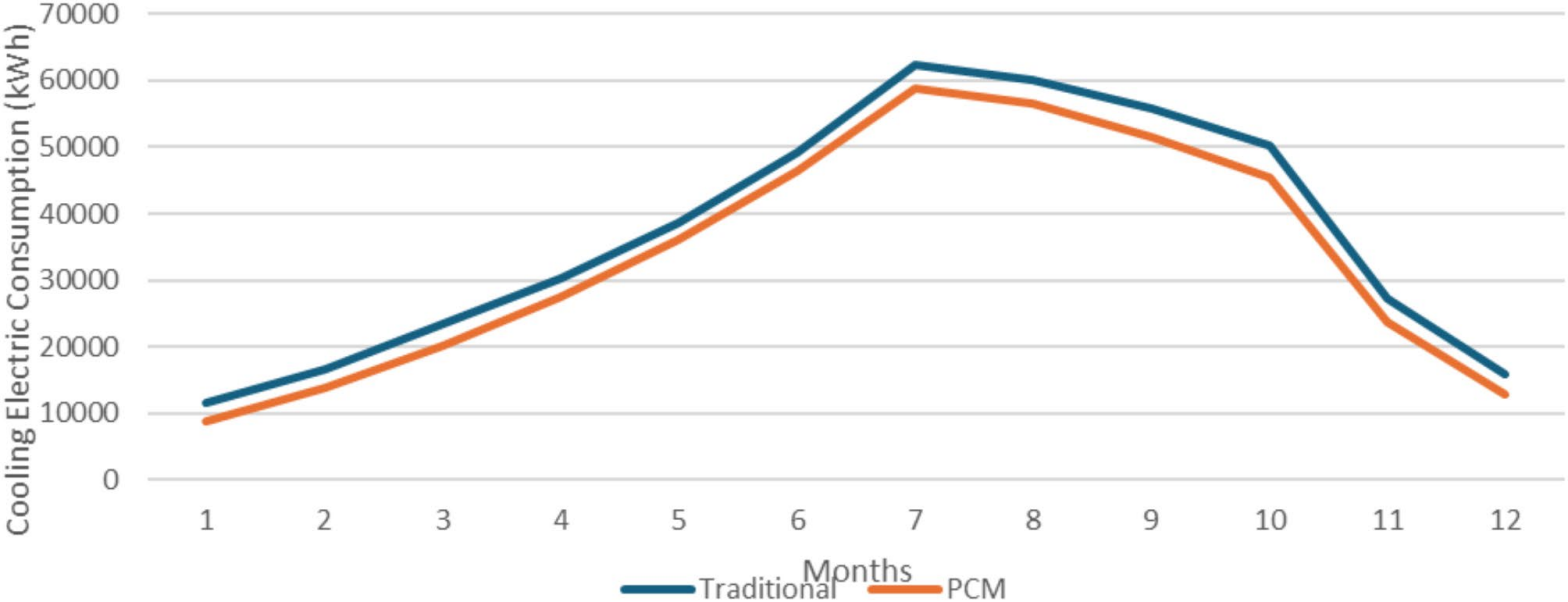
Monthly cooling electric consumption in Cairo.

The first studied location was the capital of Egypt, Cairo (Delta and Cairo climate zone). Figure 11 shows the monthly cooling electric consumption in Cairo as retrieved from the design builder simulation. It is clear that the office building with the integrated PCM double glazing units is more effective than the one with traditional DGUs in reducing the electric consumption required for cooling the building. The annual building energy consumption decreased from 440.93 MWh to 401.66 MWh, and according to Eq. (2) the annual building energy consumption was reduced by 8.91% in Cairo.

### Alexendria location

**Fig. 12:**
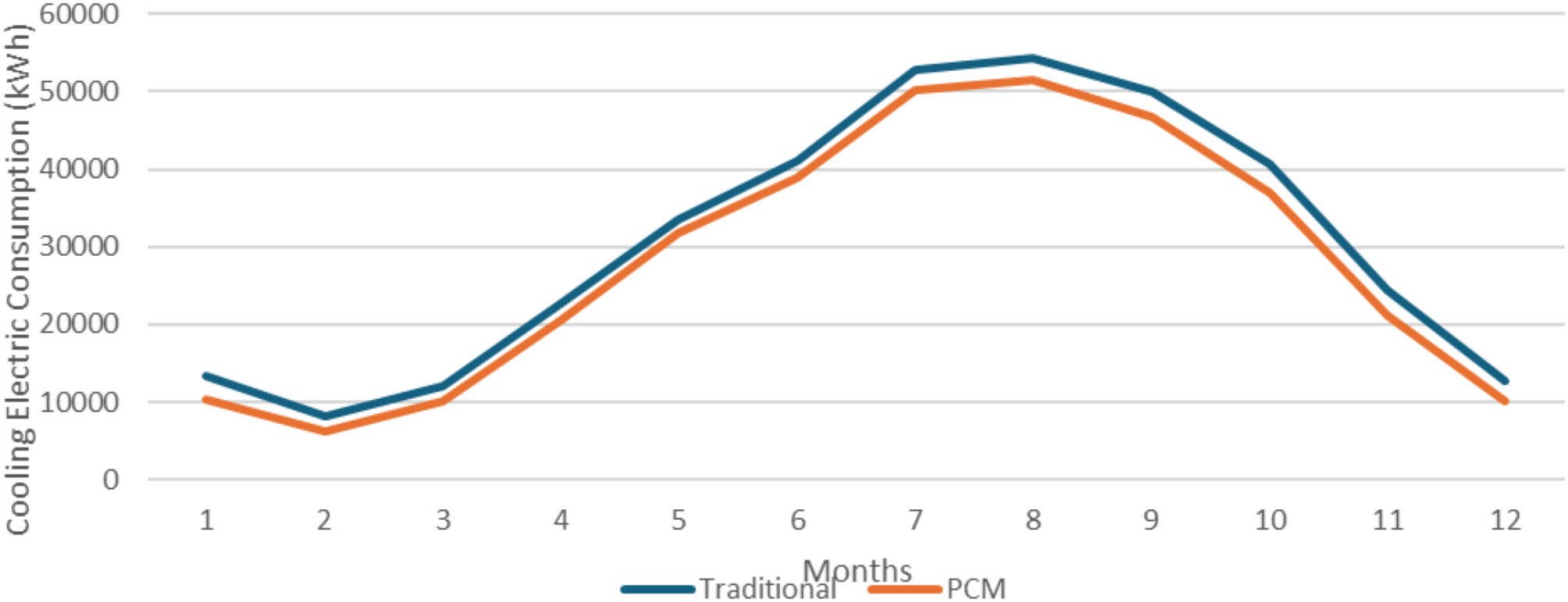
Monthly cooling electric consumption in Alexandria.

The second studied location is Alexandria (North Coast climate zone). Figure 12 shows the monthly cooling electric consumption in Alexandria as retrieved from the design builder simulation. It is clear that the office building with the integrated PCM double glazing units is more effective than the one with traditional DGUs in reducing the electric consumption required for cooling the building. The annual building energy consumption decreased from 366.01 MWh to 334.47 MWh, and according to Eq. (2) the annual building energy consumption was reduced by 8.62% in Alexandria.

### Aswan location

**Fig. 13:**
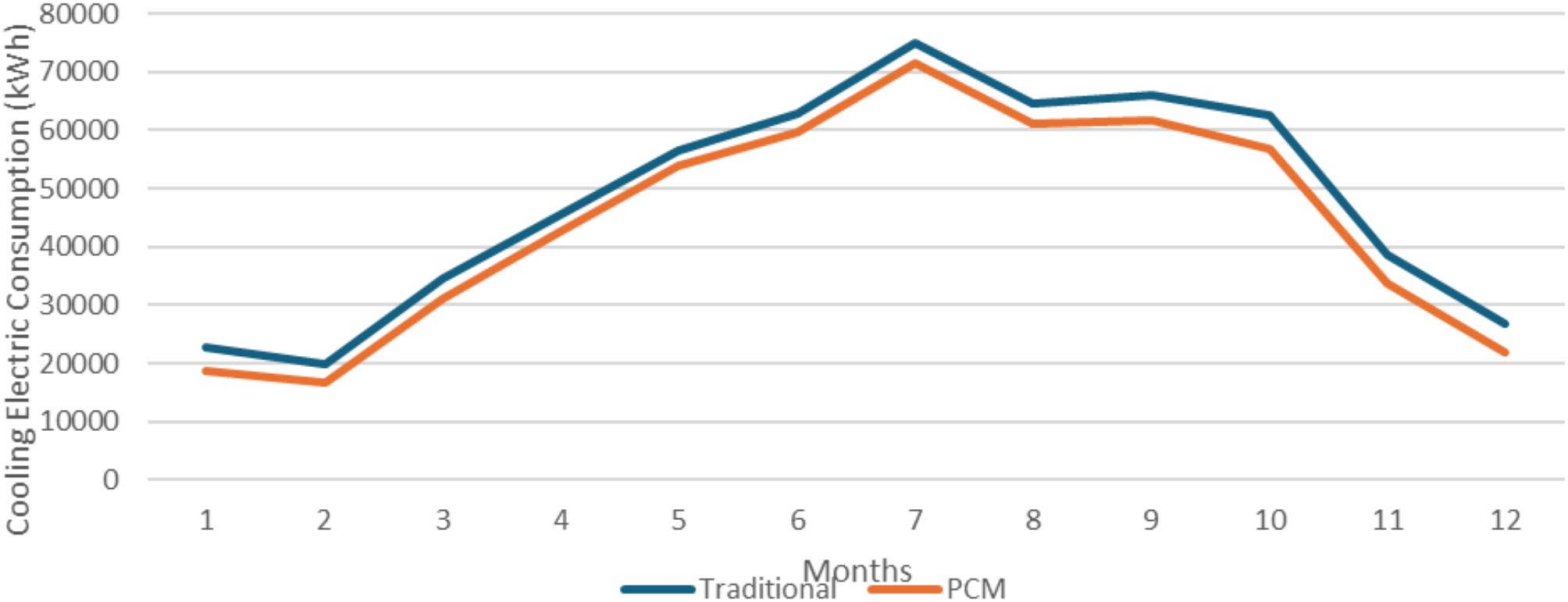
Monthly cooling electric consumption in Aswan.

The last studied location is Aswan (Southern Egypt climate zone). Figure 13 shows the monthly cooling electric consumption in Aswan as retrieved from the design builder simulation. It is clear that the office building with the integrated PCM double glazing units is more effective than the one with traditional DGUs in reducing the electric consumption required for cooling the building. The annual building energy consumption decreased from 576.02 MWh to 529.52 MWh, and according to Eq. (2) the annual building energy consumption was reduced by 8.07% in Aswan.

## Conclusions and limitations

In the current study, building thermal performance and electric energy consumption due to integrating PCM into DGUs in GCW Buildings were investigated experimentally in Cairo climate zone. After validating the simulation model using Design Builder, the office building model was then evaluated in three different climatic zones in Egypt and according to the mentioned previously operation schedule. The main findings were as follows:

Using DGUs of glass panels of 6 mm thickness and injected with PCM of 9 mm thickness have a significant effect on reducing the cooling electric energy consumption in buildings when applied to only 50% of the south and west elevations.

- The yearly cooling electric energy consumption decreased by 39.27 MWh in Cairo, which represents 8.91% from the total yearly cooling electric consumption.
- In Alexandria, the yearly cooling electric energy consumption decreased by 31.54 MWh, which represents 8.62% from the total yearly cooling electric consumption.
- In Aswan, the yearly cooling electric energy consumption decreased by 26.5 MWh, which represents 8.07% from the total yearly cooling electric consumption.

**Fig. 14:**
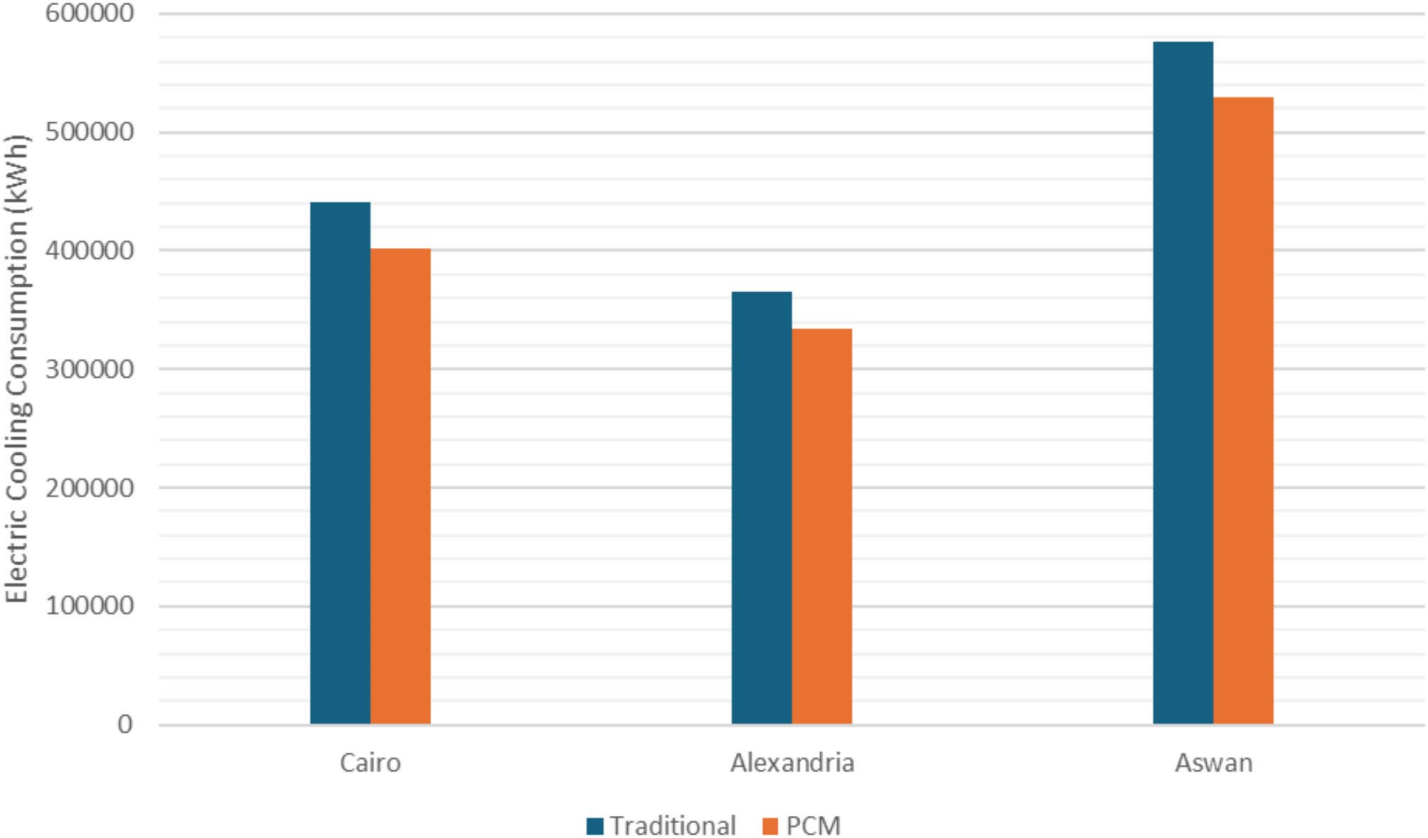
Yearly Cooling Electric Consumption in Cairo, Alexandria, and Aswan.

Finally, the annual energy consumption reduction achieved by using this technology in Egypt region ranges from 8 to 9%, and the best total energy consumption reduction when using Vaseline as PCM with DGU was in warm-tempered zones. While the least effective was when used in hot arid climatic zones in Egypt. (Fig. 14)

### Limitations

Although this study reveals the significant effect of using PCM with DGU in GCW buildings, several limitations should be mentioned:

The study is limited to the specs of the DGU of 6 mm clear glass panels and 9 mm injected PCM that was experimentally validated, any change in the DGU specs needs to be validated before applying the simulation.

It is important to mention that this study was conducted for an office building with an operation schedule of five working days a week and working hours from 08:00 to 18:00 each day, any changes to this schedule will lead to a change in the results.

Although the low cost and the availability of the used PCM (Petroleum Jelly), the study did not consider the cost of applying PCM to the DGUs, so future works should consider calculating the economic effect and the payback period of applying such technology and whether it’s feasible or not.

The study examined applying the PCM double glazing units to the south and west elevations only, so applying the PCM to the four building elevations could lead to an improvement in the building energy consumption but still its economic effect needs to be studied.

This study used the DGUs injected with PCM with only 50% of the studied elevations, increasing this percentage could lead to a better result in the building energy consumption but will come with more blocking of the view for the buildings users, this is due to the PCM used is translucent at its solid state.

The study did not consider the effect on the natural daylighting inside the building due to applying the studied PCM glazing panels. Further studies should be applied to study the effect of applying such panels on the artificial lighting energy consumption in buildings.

In further work, various cross-sections of DGU with different PCMs types should be examined. Also integrating Vaseline as a PCM within skylights could show a promising performance in reducing energy consumption in buildings. Finally, calculating the payback period would play a key role in using such glazing technology.

## Data Availability

The datasets used and/or analyzed during the current study are available from the corresponding author on reasonable request.
